# Quick Sequential Organ Failure Assessment (qSOFA) and Performance Status Scoring Systems as Prognostic Predictors in Pneumococcal Community-Acquired Pneumonia

**DOI:** 10.7759/cureus.73201

**Published:** 2024-11-07

**Authors:** Shuichi Abe, Dhammika Leshan Wannigama

**Affiliations:** 1 Department of Infectious Diseases and Infection Control, Yamagata Prefectural Central Hospital, Yamagata, JPN

**Keywords:** community-acquired pneumonia, performance status, pneumococcal, prognosis, quick sofa, scoring systems, streptococcus pneumoniae

## Abstract

Background and aim

*Streptococcus pneumoniae*, or pneumococcus, is one of the most common pathogens responsible for community-acquired pneumonia (CAP), which can progress to sepsis and lead to severe illness. Several clinical scoring systems are widely used to assess the severity of CAP and sepsis. This study aims to evaluate the clinical factors that predict mortality in pneumococcal CAP (pCAP).

Methods

Patients eligible for this study were 16 years or older and diagnosed with pCAP at Yamagata Prefectural Central Hospital, Yamagata, Japan, between January 2012 and May 2018. pCAP was defined by the single isolation of *S. pneumoniae* from sputum or blood culture in patients with CAP. Patients were divided into two groups based on 60-day mortality: survivors and non-survivors. Clinical parameters, including performance status (PS), were assessed for both groups. Disease severity was evaluated using the A-DROP, CURB-65, and quick Sequential Organ Failure Assessment (qSOFA) scores. Statistical analysis was performed using JMP 11 software (JMP Statistical Discovery LLC, NC, USA).

Results

A total of 192 patients (135 men and 57 women) were enrolled in this study. The median age was 77 years (range: 21-97 years). Among them, 169 patients were survivors and 22 were non-survivors. *S. pneumoniae* was more frequently detected in the blood cultures of non-survivors than survivors (27.3% vs. 7.7%, respectively; p = 0.01, chi-square test). Non-survivors exhibited poorer PS (PS ≥3), higher A-DROP scores (A-DROP ≥3), and higher qSOFA scores (qSOFA ≥2) compared to survivors (p = 0.002, 0.02, and 0.0003, chi-square test, respectively). However, there was no significant difference in the CURB-65 score between the two groups. Multivariate analysis revealed that higher qSOFA scores and poor PS were independent risk factors for 60-day mortality in pCAP (OR 4.0 (95% CI: 1.3-13.3) and 4.0 (1.4-10.9), respectively).

Conclusions

The qSOFA and PS scoring systems may be useful in predicting the prognosis of pCAP.

## Introduction

Pneumococcal community-acquired pneumonia (pCAP) is a major global health concern, particularly among older adults and individuals with underlying health conditions [1-3]. The causative agent, *Streptococcus pneumoniae*, can lead to severe complications such as sepsis, contributing to increased mortality rates [4-6]. Accurate risk stratification is essential for effective management of pCAP [4,5]. In Japan, the aging population has resulted in a rising incidence of pneumonia among elderly patients, especially in rural areas [4,7].

Several clinical scoring systems, including A-DROP, CURB-65, and qSOFA, are widely used to assess the severity of CAP and predict patient outcomes [3-5,8,9]. A-DROP is a system specifically designed for pneumonia patients, incorporating six key parameters: age, dehydration, respiratory rate, onset of mental confusion, and blood pressure. Each parameter is assigned points, with higher scores indicating a greater risk of adverse outcomes. This system helps clinicians determine the appropriate level of care, such as whether to admit the patient to the hospital or manage them on an outpatient basis [2,4,10]. CURB-65 evaluates five criteria: confusion, urea level, respiratory rate, blood pressure, and age (≥65 years) [2,4,7]. Higher scores correlate with increased mortality risk, guiding decisions on hospitalization and treatment intensity. Quick Sequential Organ Failure Assessment (qSOFA) is used to identify patients at risk of deterioration outside the intensive care setting. It focuses on three criteria: altered mental status, respiratory rate, and systolic blood pressure. A qSOFA score of 2 or higher suggests a higher likelihood of poor outcomes, prompting further evaluation and potential intervention [4,11,12].

While these scoring systems provide valuable frameworks for assessing CAP severity and guiding treatment, there is a notable gap in research focusing specifically on the mortality predictors for pCAP. Most studies generalize CAP without exploring the unique aspects of pCAP. This is particularly critical in ED settings, where timely decision-making and early identification of high-risk patients can significantly affect outcomes [4,6,12]. Factors such as age, comorbidities (e.g., diabetes and chronic respiratory diseases), sepsis, and laboratory findings (e.g., leukocyte count and blood gas analysis) may contribute significantly to mortality risk. Furthermore, generalized scoring systems like CURB-65 may overlook pCAP-specific factors such as bacteremia, pneumococcal serotype, and localized resistance patterns.

This study aims to explore clinical parameters alongside established scoring systems to identify potential predictors of 60-day mortality in pCAP patients. By conducting a comprehensive assessment of clinical characteristics and the applicability of scoring systems, this research seeks to enhance risk stratification and inform clinical management strategies, ultimately improving patient outcomes in this vulnerable population.

Some content of this article was previously presented as a meeting abstract at the 2018 Asian Pacific Society of Respirology (APSR) Annual Scientific Meeting on November 29, 2018.

## Materials and methods

Study design and setting

This retrospective cohort study was conducted at Yamagata Prefectural Central Hospital, Yamagata, Japan, focusing on patients diagnosed with pCAP between January 2012 and May 2018. The study was approved by the hospital’s ethics committee (COX13/2018), and informed consent was obtained from all participants.

Participants

Patients aged 16 years and older who were diagnosed with pCAP were included in the study. pCAP was defined as the isolation of *S. pneumoniae *from sputum or blood cultures in patients with CAP.

Data collection

Clinical parameters, including demographic information, underlying medical conditions, performance status (PS) [13], and laboratory test results, were collected. The PS was assessed using a scale where 0 indicates fully active and 4 indicates completely disabled. Disease severity was evaluated using the A-DROP scoring system (assessing age, dehydration, respiratory rate, orientation, and blood pressure) [14], the CURB-65 score (considering confusion, urea levels, respiratory rate, blood pressure, and age) [15], and the qSOFA score (evaluating respiratory rate, altered mentation, and hypotension).

Statistical analysis

Patients were divided into two groups based on 60-day mortality: survivors and non-survivors. Statistical analyses were conducted using JMP 11 software (JMP Statistical Discovery LLC, NC, USA). Chi-square tests were used to compare categorical variables, while multivariate logistic regression was applied to identify independent risk factors for mortality. A p-value of <0.05 was considered statistically significant.

## Results

A total of 192 patients (135 men and 57 women) were enrolled in the study (Table 1). The median age was 77 years (range: 21-97 years). Of the participants, 169 were classified as survivors and 22 as non-survivors. All non-survivors (100%) were admitted to the hospital, compared to 81.7% of survivors (p = 0.0039), indicating that hospital admission rates were higher among non-survivors. The median length of hospital stay was similar between the two groups, with survivors staying for nine days (IQR: 5-18) and non-survivors staying for 8.5 days (IQR: 2.5-26.8). Notably, *S. pneumoniae* was significantly more frequently isolated from blood cultures of non-survivors compared to survivors (27.3% vs. 7.7%, p = 0.01).

**Table 1:** Comparison of patient characteristics between the survivors and the non-survivors groups Chi-square tests were used to compare categorical variables.

Characteristics	Survivors (n = 169)	Non-survivors (n = 22)	p-value	Chi-square
Age, year, median (IQR)	75 (65.5-84)	81.5 (75.3-87)	0.0492	3.8774
Male/female, n (%)	118/51 (69.8/30.2)	17/5 (77.2/22.7)	0.4605	0.545
Hospital admission, n (%)	138 (81.7)	22 (100)	0.0039	8.329
Length of treatment, day, median (IQR)	9 (5-18)	8.5 (2.5-26.8)	0.9852	0.0004
Positive blood cultures, n (%)	13 (7.7)	6 (27.3)	0.0121	6.298

The laboratory values of survivors and non-survivors reveal several trends, although not all differences were statistically significant (Table 2). The WBC was slightly higher in survivors (13,200 ± 6,901/µL) compared to non-survivors (11,250 ± 6,307/µL), but this difference was not significant (p = 0.264). Similarly, neutrophil counts were higher in survivors (11,457 ± 6,780/µL) than in non-survivors (9,216 ± 5,776/µL), although this did not reach statistical significance (p = 0.1399). Platelet counts were nearly identical between the two groups, with survivors showing 21.4 × 10⁴/µL and non-survivors 20.3 × 10⁴/µL (p = 0.7861). CRP levels were slightly higher in non-survivors (16.1 mg/dL) compared to survivors (13.4 mg/dL), but this difference was not statistically significant (p = 0.1496). However, creatinine (Cr) levels were significantly higher in non-survivors (1.97 mg/dL) than in survivors (1.14 mg/dL) (p = 0.0104), suggesting that elevated Cr may be associated with poorer outcomes. Albumin levels were also significantly lower in non-survivors (2.8 g/dL) compared to survivors (3.3 g/dL) (p = 0.001), indicating that hypoalbuminemia may be linked to increased mortality.

**Table 2:** Comparison of laboratory testing between the survivors and the non-survivors groups Chi-square tests were used to compare categorical variables.

Laboratory parameter	Survivors (n = 169)	Non-survivors (n = 22)	p-value	Chi-square
White blood cell (/µL)	13,200 ± 6,901	11,250 ± 6,307	0.264	1.2525
Neutrophil count (/µL)	11,457 ± 6,780	9,216 ± 5,776	0.1399	2.186
Platelet count (/µL)	21.4 × 104	20.3 × 104	0.7861	0.0749
CRP (mg/dL)	13.4	16.1	0.1496	2.0825
Creatinine (mg/dL)	1.14	1.97	0.0104	6.5805
Albumin (g/dL)	3.3	2.8	0.001	10.8787

**Figure 1:**
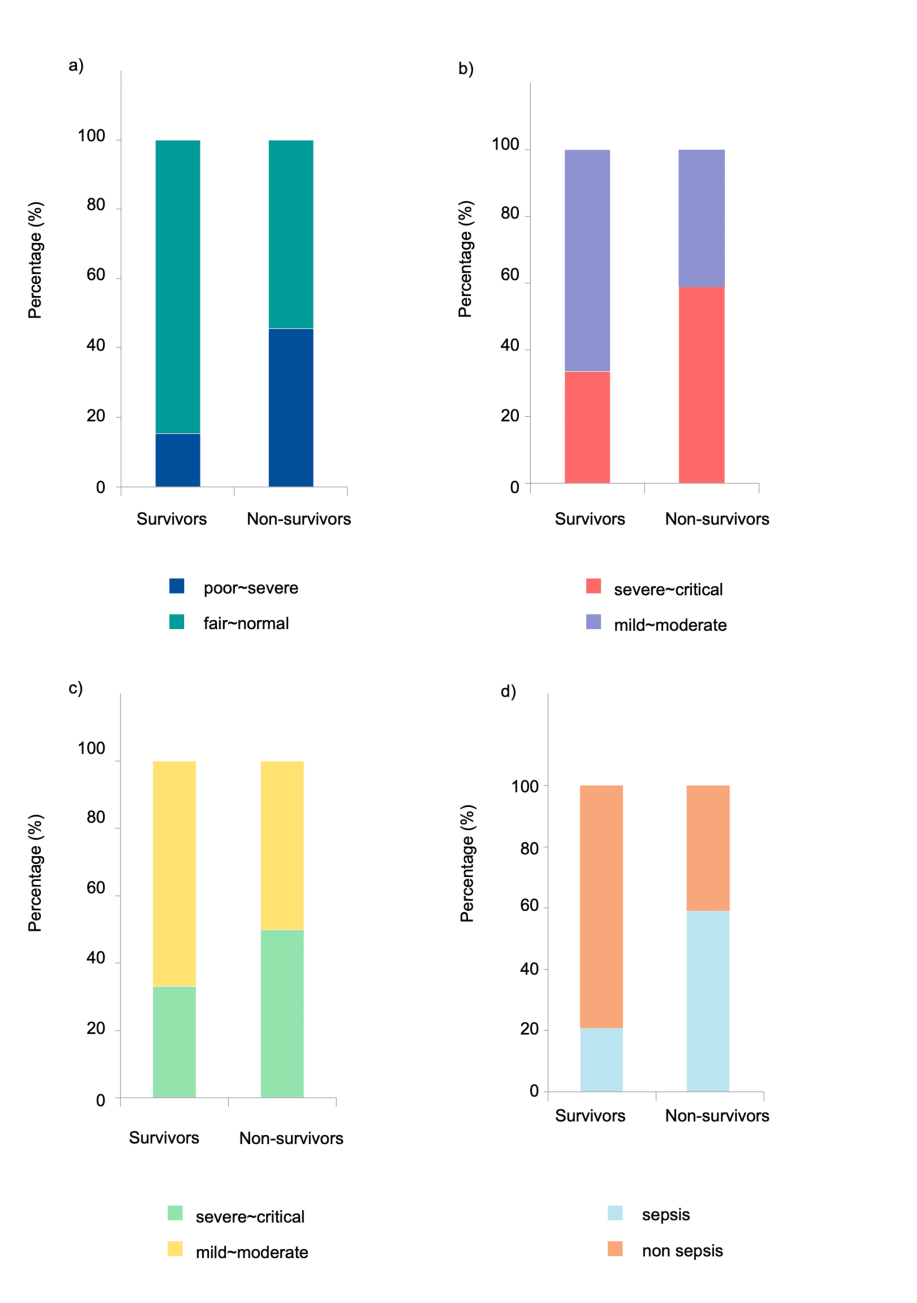
Comparison of PS, A-DROP, CURB-65, and qSOFA scores between the survivors and the non-survivors groups: (a) PS, (b) A-DROP, (c) CURB-65, and (d) qSOFA Chi-square tests were used to compare categorical variables. PS, performance status; qSOFA, quick Sequential Organ Failure Assessment

The non-survivor group exhibited poorer PS (PS ≥3), higher A-DROP (A-DROP ≥3), and higher qSOFA scores (qSOFA ≥2) compared to the survivor group, with respective p-values of 0.002 (chi-square: 9.466), 0.02 (chi-square: 13.182), and 0.0003 (chi-square: 5.176) (Figure 1a-1d). However, no significant difference was found in CURB-65 scores between the two groups (p = 0.1269, chi-square: 2.335).

Multivariate analysis identified higher qSOFA scores and poor PS as independent risk factors for 60-day mortality in pCAP, with an OR of 4.0 (95% CI: 1.3-13.3) for qSOFA and 4.0 (95% CI: 1.4-10.9) for PS (Table 3).

**Table 3:** Independent risk factors that predict disease mortality by multivariate analysis Multivariate logistic regression was employed to identify independent risk factors for mortality. PS, performance status; qSOFA, quick Sequential Organ Failure Assessment

Risk factor	OR	95% CI	p-value
Positive blood cultures	3	0.9-9.7	0.0719
High PS (≥2)	4	1.4-10.9	0.0067
High A-DROP (≥3)	1.04	0.3-3.3	0.9458
High qSOFA (≥2)	4	1.3-13.3	0.0195

## Discussion

The results of this study underscore the importance of specific clinical factors in predicting mortality among patients with pCAP. Non-survivors were more likely to have a poor PS (≥3), suggesting greater frailty, comorbidities, or overall clinical deterioration. This highlights the value of considering functional capacity alongside traditional clinical measures in assessing mortality risk. Notably, higher qSOFA scores (qSOFA ≥2) and poor PS emerged as strong predictors of mortality, emphasizing the need for early identification of high-risk patients to guide management strategies effectively.

In addition to qSOFA, A-DROP scores (A-DROP ≥3) were significantly more prevalent among non-survivors, reinforcing the utility of these scoring systems in identifying high-risk patients [16]. Despite the widespread use of CURB-65 in assessing CAP severity, our results suggest it may be less effective in predicting mortality for pCAP, while qSOFA and A-DROP scores successfully differentiated survivors from non-survivors. This indicates that alternative scoring tools, such as qSOFA and PS, might offer more reliable prognostic information [4]. Incorporating these tools into clinical practice could improve patient outcomes, particularly in high-risk groups, by enabling timely interventions. Our findings align with previous studies that demonstrate a correlation between SOFA scores and pneumonia mortality [17]. These results highlight the significance of integrating functional assessments and qSOFA scoring into routine clinical evaluations for pCAP, offering superior prognostic value compared to traditional scoring systems like CURB-65 [9,11,12].

Moreover, the significantly higher prevalence of *S. pneumoniae* in blood cultures from non-survivors suggests that bacteremia, a marker of more severe infection, is closely linked to poor outcomes in pCAP [3,4,6]. While bacteremia detection may serve as an important prognostic indicator, it alone may not be sufficient to predict mortality.

However, this study is not without limitations. Its retrospective design and small sample size, particularly among non-survivors, may limit the generalizability of the findings. Larger, prospective studies are necessary to validate these results. Future research should also explore the contributions of other clinical and laboratory parameters, such as inflammatory markers or co-infections, to refine pCAP risk stratification.

This study emphasizes the need for a multifaceted approach to pCAP risk assessment, incorporating both functional status (e.g., PS) and scoring tools like qSOFA. Tailoring treatment and monitoring strategies based on these factors could optimize outcomes for this high-risk patient population. Future studies should investigate whether integrating bacteremia detection with scoring systems like qSOFA into clinical management algorithms can enhance prognostic accuracy and resource allocation, potentially reducing mortality in this vulnerable group.

## Conclusions

This study highlights the significance of incorporating qSOFA and PS as reliable predictors of mortality in patients with pCAP. By integrating these scoring systems into routine clinical practice, healthcare providers can improve risk stratification, leading to more accurate assessments of patient prognosis. This approach allows for the early identification of high-risk individuals and the implementation of tailored management strategies, ultimately improving patient outcomes and optimizing the allocation of healthcare resources. The findings highlight the potential of these tools to enhance decision-making and elevate the quality of care in the management of pneumococcal pneumonia.
